# Worries during the COVID-19 pandemic – which were the most prevalent and disturbing?

**DOI:** 10.1192/j.eurpsy.2022.504

**Published:** 2022-09-01

**Authors:** A. Manão, A.T. Pereira, C. Cabacos, A.P. Amaral, M.J. Soares, A. Macedo

**Affiliations:** 1 Faculty of Medicine of University of Coimbra, Institute Of Psychological Medicine, Coimbra, Portugal; 2 Centro Hospitalar e Universitário de Coimbra, Department Of Psychiatry, Coimbra, Portugal; 3 University Beira Interior, Faculty Of Health Sciences, Covilhã, Portugal; 4 Coimbra Institute for Biomedical Imaging and Translational Research, Icnas, Coimbra, Portugal; 5 University of Beira Interior, Faculty Of Health Sciences, Covilhã, Portugal

**Keywords:** covid, health, Covid-19 pandemic, contamination

## Abstract

**Introduction:**

The COVID-19 pandemic has brought additional worries and challenges to people’s lives, with potential implications for psychological well-being.

**Objectives:**

To understand which worries and life changes have affected most the Portuguese general population during the COVID-19 pandemic and to analyse which contents are associated with higher levels of repetitive negative thinking/RNT and psychological distress/PD.

**Methods:**

In September-December 2020, 413 Portuguese adults (69.2% female; Mean age= 31.02±14.272) were asked one open questions, with reference to the COVID-19 pandemic period: “what was your biggest worry?”; the answers were independently categorized by two researchers. Participants also filled the validated Depression Anxiety and Stress Scale and the Perseverative Thinking Questionnaire.

**Results:**

The most prevalent worries were about: 1) fear of contamination (oneself and others-48.7%; 2) physical and mental health and well-being (self and others)-27.2%; 3) studies and profession-13.3%; 4) uncertainty about the future-7.7%; 5) economic-financial issues-6.5%; 6) miscellaneous-3.3%; 7) no worries-0.7%. Participants who had worries of the theme 4 had the highest RNT and PD mean scores, followed by themes 3 and 5, and then themes 2 and 1. These thematic groups significantly (p<.01) differ between each other (except 3-5) and from the other groups. RNT was a significant predictor of PD (R^2^=37.0%, β=.609, p<.001).

**Conclusions:**

People who worry about the future uncertainties, occupational activities and finances should be systematically assessed with regard to their levels of anxiety, depression and stress and they can learn to deal with the RNT as a way to reduce their psychological suffering in times of pandemic.

**Disclosure:**

No significant relationships.

